# Patient-focussed outcomes in acromegaly

**DOI:** 10.1007/s11102-013-0519-8

**Published:** 2013-09-20

**Authors:** Mirtha Guitelman, Alin Abreu, Ana Laura Espinosa-de-los-Monteros, Moisés Mercado

**Affiliations:** 1 División Endocrinología, Hospital Carlos G. Durand, Buenos Aires, Argentina; 2 Endocrinología, Centro Médico Imbanaco, Cali, Colombia; 3 Servicio de Endocrinología, Hospital de Especialidades, Centro Médico Nacional S.XXI, IMSS, Mexico City, Mexico; 4 Faculty of Medicine, Universidad Nacional Autónoma de México, Mexico City, Mexico; 5 Endocrine Service, Experimental Endocrinology Unit, Hospital de Especialidades, Centro Médico Nacional Siglo XXI, Instituto Mexicano del Seguro Social, Aristóteles 68 Polanco, 11560 Mexico City, Mexico

**Keywords:** Acromegaly, Pituitary, Latin America, GH, IGF-1, Quality of life, Somatostatin analogs

## Abstract

**Background:**

Health-related quality of life (QoL) is severely impaired in acromegaly due to the physical and psychological consequences of the disease. Pharmacological and surgical treatments, when available, can improve QoL and life expectancy.

**Case description:**

A 34-year-old male with uncontrolled acromegaly due to a large and invasive macroadenoma, which could not be resected by transsphenoidal surgery. Over 9 years, he had limited access to pharmacological interventions and persisted with clinically and biochemically active disease, with severe co-morbidities and a poor QoL, which eventually lead to a premature sudden death.

**Conclusion:**

This case highlights the impact that active acromegaly has when treatment resources are limited. We review the factors contributing to poor QoL in this disease, with special reference to the Latin American scenario.

## Introduction

Health-related quality of life (QoL) is known to be severely impaired in patients with acromegaly [1–3]. Several validated tools have been used to assess QoL in patients with acromegaly, including one disease-specific instrument called the acromegaly quality of life questionnaire (AcroQoL), which involves 22 items concerning the physical dimension of the disease and two separate psychological dimensions connected with appearance and personal relations [4, 5]. Although improvements in QoL can be achieved with pharmacological and surgical interventions, physical and psychological residual morbidity and worse coping strategies can lead to persistently impaired QoL, even in acromegalic patients with long-term biochemical remission [2, 3, 6–11]. As such, QoL considerations in both treated and untreated patients are important for the long-term management of acromegaly. In this article, we provide a brief overview of the factors contributing to poor QoL in acromegaly, with reference to a Latin American case study, and discuss how QoL can be improved.

**Fig. 1:**
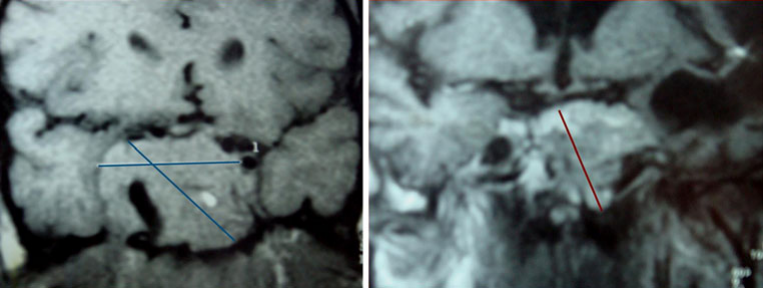
MRI scans of pituitary tumor at diagnosis (December 1994, *left*) and 9 years after surgery and radiotherapy without medical treatments (January 2008, *right*)

## QoL in active and controlled acromegaly

The available evidence from both disease-specific and generic QoL tools consistently shows that patients with acromegaly (even those in biochemical remission) have reduced QoL compared to matched populations without acromegaly [1, 2, 5, 6, 8, 12, 13]. The most affected aspects of QoL in acromegaly are related to appearance and the least affected are related to personal relations [2, 5, 14].

Several factors appear to predict worse QoL in acromegaly, including disease activity, female gender, older age, longer disease duration, the presence of specific symptoms/comorbidities, certain treatment modalities and delays in diagnosis [2, 5–7, 15]. In terms of disease activity, some (but not all) studies have shown that patients in biochemical remission have significantly better QoL (at least in some dimensions) than patients with persistent disease, or that improvements in biochemical parameters correlate with improved QoL [1, 5, 8, 11, 12, 14, 16–19]. However, it should also be noted that patients with acromegaly who develop growth hormone deficiency (GH) after treatment also have decreased QoL [20].

Several disease-specific symptoms or comorbidities of acromegaly are associated with poor QoL. Joint-related complications (leading to pain and immobility), in particular, are frequently encountered in acromegaly and have been shown to have a significant negative impact on QoL [7, 21–25]. These complications may persist during biochemical remission and represent a major cause of morbidity [7, 23, 24]. Sleep apnea syndrome is another common complication of acromegaly that has a negative influence on QoL [11, 26]. Other comorbidities such as depressive mood and hypertension may also be associated with poor QoL in acromegaly [11, 22].

Among treatment modalities, radiotherapy in particular has been associated with reduced QoL [7, 27]. In one study that followed patients with stable biochemical control over 4 years, previous radiotherapy was the predominant predictor of progressive impairments in QoL [27]. This may relate to the effects of GH deficiency, among other deficiencies, that can occur with definitive therapy for acromegaly using irradiation or surgery [3, 20].

**Table:** Case study: 9 years of uncontrolled disease without appropriate medical therapy: a case for poor QoL (Mirtha Guitelman, MD)

A 34-year old man presented with headaches, acral enlargement, oily skin, sleep apnea, fatigue, hyperhidrosis and generalized joint pain
All these symptoms started 5 years prior to the first consultation
The patient had no relevant background
Physical exam revealed
Thyroid: 30 g
Typical facial features: lip, nose and tongue enlargement
Prognathism
Acral enlargement (finger size: 33)
No Hypertension
Endocrinological lab results (Nov 1993)
Baseline and post-glucose GH 50 μg/L
IGF-1: 700 μg/L (normal for age ≤500 μg/L)
Prolactin: 200 μg/L (normal 5–20 μg/L)
Total T4: 1.2 nmol/L (6.9 μg/dL); TSH: 1.6 mIU/L
Testosterone: 6.6 nmol/L (190 ng/dL)
LH: 3 IU/L; FSH: 2.8 IU/L
Normal glucose metabolism
MRI at diagnosis (Dec 1994)
Voluminous sellar mass with sphenoid sinus invasion, extension in the suprasellar cistern causing displacement of the pituitary stalk and invasion into the right cavernous sinus (Fig. 1)
Complementary studies
Echocardiogram: Mild dilated left ventricle with appropriate systolic function. Left and right ventricular dilatation
Treatments
In 1995 he received intermittently subcutaneous SSAs 300 μg/day and bromocriptine for 6 months with no changes in IGF-1 levels or tumor size
Due to the lack of drug availability and lack of response, the patient was sent to surgery
Transsphenoidal surgery (Aug 1996)
Pathology: GH-Prolactin co-secreting tumor
GH after surgery: 51 μg/L
Transcranial surgery (Apr 1997)
GH after surgery: >30 μg/L
Hypogonadotrophic hypogonadism
Normal thyroid and adrenal function
Radiotherapy 5,000 CGy (May 1998)
One year after radiotherapy
IGF-1: >400 μg/L
GH: 40 μg/L
Prolactin: 80 μg/L
Rest of anterior pituitary hormones normal
Bromocriptine and somatostatin analogs were indicated
The patient returned (Jan 2008) after 9 years without any treatment
Joint stiffness, paresthesia, arthropathy (osteoarthritis, needed a walking stick), macroglossia, severe obstructive sleep apnea and headache
Acral enlargement, increased sweating
Signs and symptoms of Hypopituitarism
Hormonal evaluation after 9 years without treatments
GH: 19.9 μg/L; IGF-1: 640 μg/L (normal for age 101–303 μg/L)
Prolactin: 860 μg/L
LH: 0.2 IU/L; FSH: 0.5 IU/L; Testosterone: 0.3 nmol/L (0.1 ng/mL)
Cortisol: 28 nmol/L (1 μg/dL)
Free T4: 8.4 pmol/L (0.65 ng/dL); TSH: 2.7 mIU/L
*Active disease plus complete Hypopituitarism*
For MRI scan in Jan 2008, 9 years after surgery and radiotherapy without medical treatments, see Fig. 1
Treatment indications
Hydrocortisone 15 mg/day
L-T4 100 μg/day
Testosterone Transdermic testosterone 5 g, one sachet/day
Cabergoline 1 mg/week
SSA
Follow-up
The patient didn’t return to the hospital again after February 2008, in spite of attempts to contact him for follow-up
The patient died suddenly in January 2010 at age of 49
At the time of death, he had been on regular L-T4, hydrocortisone and cabergoline, as well as irregular testosterone replacement
He never received somatostatin analogs due to difficulties obtaining the drug

## Case discussion

This 49-year-old man with acromegaly diagnosed at age 34, never achieved normal levels of IGF-1 and GH despite two surgeries and radiotherapy after initially receiving suboptimal pharmacological therapy. He subsequently received no treatment at all over a period of 9 years followed by further suboptimal pharmacological therapy and died early due to lack of disease control. Patients with acromegaly have significantly increased mortality, even after transsphenoidal surgery [28]. However, achievement of biochemical control has been shown to significantly improve life expectancy [29].

Apart from the patient’s reduced life span another important aspect of the patient’s poor disease control was the severe debilitating complications of acromegaly that he had to endure for many years. As a consequence, it is highly likely that this patient experienced profound long-term impairments in QoL derived from his multiple systemic complications. The presence of severe arthropathy, in particular, is known to be a major cause of morbidity and immobility in acromegaly and has a marked negative impact on QoL [24, 25]. Other factors, such as sleep apnea and psychological aspects associated with his acromegalic appearance may also have reduced the patient’s QoL. The patient is also an illustrative example of the consequences of a delayed diagnosis; had his condition been detected earlier, he would have undoubtedly had a different and better outcome.

Due to the invasive nature of this patient’s tumor, pharmacological therapy with SSAs would have been the primary treatment of choice, but these agents were only available intermittently for a short period after diagnosis and this option could not be implemented appropriately. The question is whether long-term pharmacological treatment with SSAs or other pharmacological therapies could have changed the QoL of this patient, as well as improving his life expectancy.

## Improving QoL in acromegaly

As noted above, there is some evidence to suggest that patients with biochemically controlled acromegaly have significantly better QoL (at least on some subscales) than patients with persistent disease, although not all studies have found a strong association [1, 5, 8, 11, 12, 14, 16–19]. One study suggests that the best QoL in treated patients seems to be achieved if insulin-like growth factor-1 (IGF-1) is normalized and the GH nadir (during an oral glucose tolerance test) is targeted to levels less than 1.0 μg/L but above 0.3 μg/L [8].

Several studies have also shown consistently that treatment with surgery or long-acting SSAs is associated with significant improvements in QoL [30–35]. This might be expected, as these treatments can provide significant improvements in symptoms known to be associated with poor QoL, especially in the frequency and severity of sleep apnea episodes [26]. With the exception of radiotherapy, which is generally associated with poorer QoL, it is hard to distinguish any notable differences on overall QoL between different treatment modalities [14]. Randomized comparative studies in newly diagnosed patients suggest that long-acting SSAs and surgery provide similar levels of QoL when used as first-line therapy [32, 35]. Pharmacological combination therapy (e.g., adding pegvisomant to an SSA) may also provide better improvements in QoL independently of having better biochemical control [36].

It should be emphasized that, although clinically and biochemically effective, some SSAs need to be injected by a properly trained professional, and thus, patients are required to visit the hospital or medical office every few weeks. Although usually not a major problem, this may generate more psychological distress, social fears and a poorer sense of well-being and may hamper improvements in QoL [17, 37]. Clinical trial and observational study evidence suggests that patients prefer the option to administer SSAs themselves or by a partner at home rather than by a healthcare professional, and this does not appear to compromise efficacy or safety [38–42]. Further evidence also suggests that patients also prefer longer intervals between injections, and this also appears to be achievable with certain SSAs without loss of efficacy [41, 43].

It has been suggested that targeted psychosocial interventions, such as cognitive behavioral therapy, self-management training, and information on the negative effects of the disease might help biochemically controlled patients with acromegaly to use more active coping strategies, which might lead to improvements in QoL [10]. In a recent study, greater diagnostic delay by the physician (relative to a patient first seeking medical advice) was associated with reduced psychological QoL, depression, sleep disturbances and impaired body image, suggesting that improved physician awareness of acromegaly (leading to earlier diagnosis) may also help to improve QoL [15]. To our knowledge only one Latin-American study has evaluated QoL in acromegaly using an adapted version of the AcrQoL questionnaire [46]. This study from a tertiary care center in Mexico City found that the 50 patients with acromegaly have reduced scores in all scales of the AcroQol (physical, psychological, appearance and interpersonal relationships) when compared to controls [46]; yet, no differences in any of these scales were found between active and controlled patients, perhaps due to the relatively low number of patients [46].

## Conclusions

Patients with long-term uncontrolled acromegaly, as in our case study, are likely to have severe acromegalic features, symptoms and comorbidities and consequently significantly impaired QoL and reduced life expectancy. However, there are many unanswered questions about the benefits of controlled compared with uncontrolled acromegaly regarding QoL and other factors, such as costs and morbidities [14, 19]. For instance, normalization of levels of IGF-1 and GH do not necessarily reflect optimal QoL or symptom relief [44]. As such, QoL considerations in both treated and untreated patients are important for the long-term management of acromegaly and it has been suggested that simplified measures of QoL may provide a reliable additional measurement of disease activity in everyday clinical practice [44].

It is also possible that reduction in tumor mass (which may be independent of biochemical control with SSAs [45]) has the potential to improve QoL, although this remains to be further investigated. With the available evidence it is hard to distinguish any notable consistent differences in QoL between pharmacological therapy and surgery (at least in the first-line setting), whereas radiotherapy appears to be associated with a reduced QoL [14, 32, 35]. However, some factors associated with pharmacological therapy, such as regular hospital visits for drug administration, may have an adverse impact on QoL, and there is the potential to minimize this through the use of drug formulations that require less frequent dosing and/or can be administered at home without the help of a healthcare professional. Longer-acting drugs may also have the potential to improve QoL through tumor shrinkage. Prompt diagnosis and targeted psychosocial interventions may also be an important factors leading to better QoL.

In summary, in patients with active acromegaly, as well as those in remission, attention to QoL issues is highly recommended [3]. The best balance of efficacy, cost and QoL will likely be achieved with an individualized approach to therapy, based on available pharmacological, surgical and radiotherapeutic resources [14].
